# Dysregulation of HO-1-SIRT1 Axis is Associated with AngII-Induced Adipocyte Dysfunction

**Published:** 2024-05-20

**Authors:** Hari Vishal Lakhani, Mishghan Zehra, Sneha Pillai, Joseph I. Shapiro, Komal Sodhi

**Affiliations:** Department of Surgery, Internal Medicine, and Biomedical Sciences, Joan C Edwards School of Medicine, Marshall University, Huntington, United States of America

**Keywords:** Angiotensin II, Oxidative stress, Heme oxygenase 1, Sirtuin 1, Mineralocorticoid receptor, Adipocyte dysfunction

## Abstract

Angiotensin II (AngII), a component of the Renin-Angiotensin-Aldosterone System (RAAS), has been implicated in the dysregulation of adipose tissue function. Inhibition of AngII has been shown to improve adipose tissue function in mice with metabolic syndrome. It is well established that the Heme Oxygenase-1 (HO-1), an antioxidant improves oxidative stress and phenotypic change in adipocytes. Molecular effects of high oxidative stress include suppression of Sirtuin-1 (SIRT1), which is amenable to redox manipulations. However, the underlying mechanisms by which the Renin-Angiotensin-Aldosterone System (RAAS) exerts its metabolic effects are not fully understood. In this study, we propose that AngII-induced oxidative stress may suppress adipocyte SIRT1 through down-regulation of HO-1. Consequently, this suppression of SIRT1 may result in the up-regulation of the Mineralocorticoid Receptor (MR). We further hypothesize that the induction of HO-1 would rescue SIRT1, thereby improving oxidative stress and adipocyte phenotype. To establish this hypothesis, we conducted experiments using mouse preadipocytes treated with AngII, in the presence or absence of Cobalt Protoporphyrin (CoPP), an inducer of HO-1, and Tin Mesoporphyrin (SnMP), an inhibitor of HO-1. Our data demonstrate that treatment of mouse preadipocytes with AngII leads to increased lipid accumulation, elevated levels of superoxide and inflammatory cytokines (Interleukin-6 and Tumor necrosis factor alpha), and reduced levels of adiponectin. However, these effects were attenuated by the induction of HO-1, and this attenuation was reversed by SnMP, indicating that the beneficial effects on adipocyte phenotype are modulated by HO-1. Furthermore, our findings reveal that AngII-treated preadipocytes exhibit upregulated MR levels and suppressed *SIRT1* expression, which are rescued by HO-1 induction. Following treatment with CoPP and *SIRT1* siRNA in mouse preadipocytes resulted in increased lipid accumulation and elevated levels of fatty acid synthase, indicating that the beneficial effects of HO-1 are modulated through SIRT1. Our study provides evidence that HO-1 restores cellular redox balance, rescues SIRT1, and attenuates the detrimental effects of AngII on adipocytes and systemic metabolic profile.

## INTRODUCTION

The Renin-Angiotensin-Aldosterone System (RAAS) is a significant regulator of the systemic homeostasis and has been recognized as an endocrine axis that acts on several organs, with a negative feedback loop where increased levels of circulating Angiotensin II (AngII) inhibit the renal renin release [1]. Previous studies have demonstrated that the RAAS primarily regulates vascular, cardiac, and renal functions [2–4]. One of the latest additions to this expanding understanding is the recognition that Visceral Adipose Tissue (VAT), which is considered an endocrine organ, expresses, and is regulated by the RAAS [5]. Studies have shown that VAT expresses all components of the RAAS, such as Angiotensinogen, Renin, and Aldosterone Synthase (*CYP11B2*) [6–8]. Excessive VAT accumulation is associated with obesity, a global health concern affecting a significant proportion of the adult population and has also been closely related to mortality [9]. It has been shown that blocking the RAAS can reduce obesity-related cardiovascular and renal complications, improve oxidative stress, and promote adipocyte function [10–12]. In cultured mouse preadipocytes, AngII has been shown to modulate cellular metabolism, leading to a decrease in proliferation and an increase in lipid accumulation [13,14]. In addition, animal studies have demonstrated that increased AngII levels are associated with visceral adiposity and reduced plasma adiponectin levels [15,16]. Essential components of the RAAS, including *CYP11B2* and the Mineralocorticoid Receptor (MR), also influence adipocyte structure and function [17,18]. MR blockade has been found to improve cardiovascular and hepatic complications associated with adiposity and enhance adipocyte differentiation [19–22]. In mouse preadipocytes, aldosterone has been shown to stimulate *MR*-dependent adipogenesis [19]. However, the direct impact of the RAAS on VAT/adipocyte structure and function remains unresolved, and the underlying mechanisms of RAAS involvement in these processes are not yet fully understood.

It has been shown that increased VAT mass leads to inflammatory infiltration and impairs the secretion of protective adipokines, such as adiponectin. Consequently, VAT hypertrophy triggers a complex pathophysiological response characterized by systemic release of inflammatory and oxidative molecules and reduced secretion of protective adipokines. Studies have been showing that oxidative stress and chronic redox imbalance promote lipogenesis and contribute to adipocyte dysfunction [23–25]. The RAAS has been implicated in lipid accumulation, which is attributed to RAAS-induced redox imbalance [14,26]. High oxidative stress can suppress Sirtuin-1 (*SIRT1*), a key protein involved in adipogenesis and responsive to redox modulation [23,27,28]. It has been shown that the overexpression of SIRT1 can prevent redox-induced adipocyte hypertrophy and dysfunction [29,30]. In addition, it’s documented that the antioxidant properties of Heme Oxygenase-1 (HO-1) induction through CoPP improves the metabolic imbalance in high-fat diet mice [31].

Based on these observations, we aim to investigate the mechanistic link between an overactive RAAS specific to VAT and the subsequent development of adipocyte and metabolic dysfunction through the increase of cellular oxidative stress. We hypothesize that AngII-induced oxidative stress has the potential to downregulate adipocyte *SIRT1* levels by suppressing *HO-1* expression. This study will unveil the negative regulatory influence of the HO-1-SIRT1 axis, thereby improving the effects exerted by AngII on adipocyte phenotype. Consequently, the main objective of this study is to elucidate the molecular interplay among AngII, HO-1, and SIRT1 in relation to the regulation of adipocyte structure and function, ultimately impacting systemic metabolic homeostasis.

## MATERIALS AND METHODS

### Experimental design for in vitro experiments

Frozen mouse preadipocytes (3T3-L1), obtained from ATCC, were suspended in α-Minimal Essential Medium (α-MEM) supplemented with 10% inactivated fetal bovine serum and 1% antibiotic/antimycotic solution. The cell cultures were maintained at 37°C in a 5% CO_2_ incubator, with medium changes performed every 48 hours. Upon reaching 80% confluence, the cells were harvested using trypsin. Subsequently, the cells were plated in 96-well and 24-well plates at a density of 10,000 cells/cm^2^ and cultured until 80% confluence was achieved. The cells were then cultured for an additional 7 days in adipogenic medium to induce adipocyte differentiation. In the treatment groups, the cells were treated every alternate day for a duration of 7 days with AngII (10 μM), CoPP (5 μM), and SnMP (5 μM). The control groups were treated with adipogenic media alone to induce adipocyte differentiation, without any additional treatment. Additionally, an experimental group was included in which murine preadipocytes were treated with SnMP alone to assess its inhibitory effects.

For knockdown studies, commercially available (Ambion Silencer Select) siRNA specific to *SIRT1*, along with an appropriate scrambled RNA control, was employed. In the over-expression studies, the full-length variant (isoform 1, Gene ID 93759) of mouse *SIRT1* was synthesized into the pJ603 vector, along with the corresponding pJ603-GFP negative control, by DNA 2.0 Inc. The transfection of cells was achieved using the FuGENE^®^ HD transfection reagent.

### Oil Red O staining

Lipid droplets were visualized by performing Oil Red O (ORO) staining after 7 days. For this staining, a solution of 0.21% ORO in 100% isopropanol (Sigma-Aldrich, St Louis, MO, USA) was prepared. 3T3-L1 adipocytes were fixed in 10% formaldehyde for 15 minutes, followed by incubation with ORO solution for 20 minutes. The cells were then rinsed with phosphate buffered saline to remove excess stain. For qualitative study, pictures were captured randomly using a phase contract microscope (Olympus CK30). To elute ORO, 100% isopropanol was added for 10 minutes, and the optical density was measured at 490 nm for quantitative analysis.

### Cytokines, adiponectin and lipid profile measurements

Conditioned Media (CM) was collected from the cell culture. The levels of Interleukin-6 (IL-6), Tumor Necrosis Factor Alpha (TNF-α), and the High Molecular Weight (HMW) form of adiponectin were quantified using Enzyme-Linked Immunosorbent Assay (ELISA) kits following the manufacturer's protocol (Abcam, Cambridge, MA). Triglyceride levels in the conditioned media were measured using an ELISA assay kit (Assay Gate, Inc.).

### Quantitative real-time Polymerase Chain Reaction (PCR) analysis

Total RNA, extracted from mouse adipocytes using the RNeasy Protect Mini Kit (QIAGEN, Maryland, USA), was analyzed by quantitative real-time Polymerase Chain Reaction (PCR). Real-time PCR was performed on a 7500 HT Fast Real-Time PCR System (Applied biosystems) using SYBR Green PCR Master Mix. The following primers targeting HO-1, Fatty Acid Synthase (FAS), Peroxisome Proliferator-Activated Receptor Gamma Coactivator 1-Alpha (PGC-1α), IL-6, SIRT1, *CYP11B2*, MR, Angiotensin II Receptor type 1 (AT1R), and Glyceraldehyde 3 Phosphate Dehydrogenase (GAPDH) were employed. The fold amplification was calculated using the comparative threshold cycle method as specified by the manufacturer. To normalize the experimental samples, *GAPDH* was used as a house keeping gene.

### Measurement of superoxide levels for in vitro experiment

3T3-L1 mouse adipocytes cells were cultured on 96-well plates until they reached approximately 70% confluence. Subsequently, the cells were treated with AngII (10 μM) either alone or in combination with CoPP (5 μM) and SnMP (5 μM) for 2 days. Following the treatment, the cells were incubated with 10 μM Dihydroethidium (DHE) in the dark at 37°C for 30 minutes.

The fluorescence intensity was then measured using a Perkin-Elmer Luminescence Spectrometer with excitation/emission filters set at 530/620 nm.

### Statistical analysis

Statistical significance between experimental groups was determined by Tukey’s post hoc test for multiple comparison. For comparisons among treatment groups, the null hypothesis was tested by a two-factor Analysis of Variance (ANOVA). Statistical significance was assigned at p<0.05 or p<0.01 for confidence interval of 95% or 99%, respectively. Data are presented as mean ± SEM.

## RESULTS

### HO-1 improves AngII-induced phenotype alteration of adipocytes

**Figure 1:**
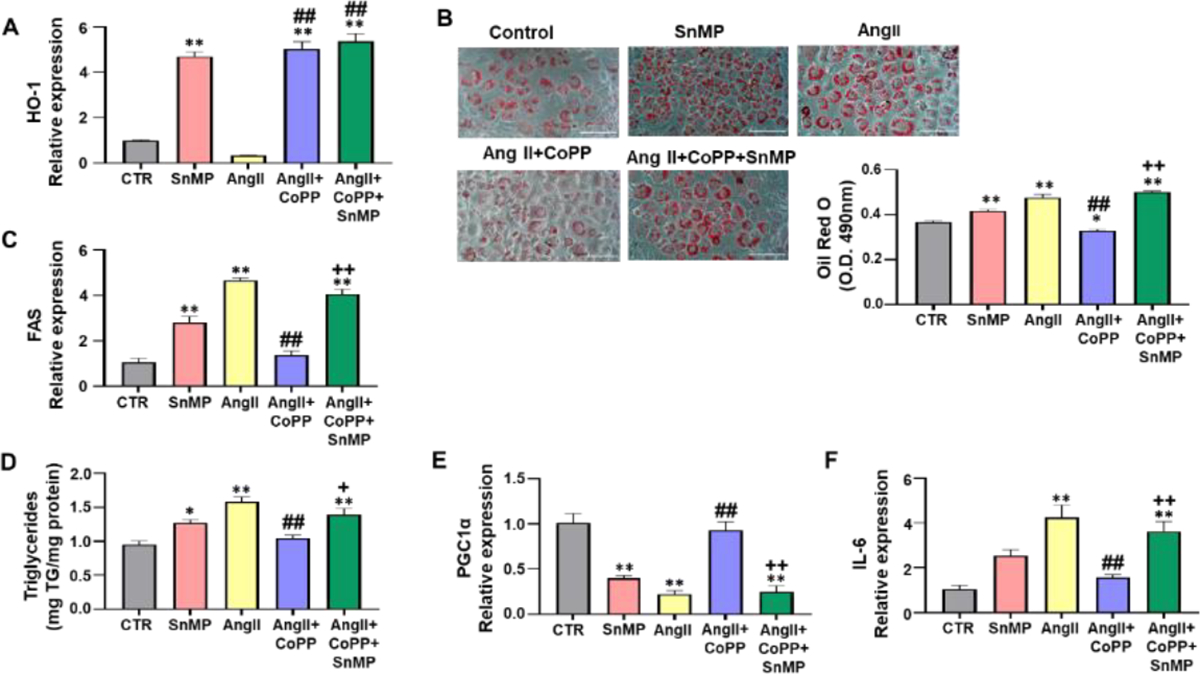
Effect of AngII exposure on 3T3-L1 murine preadipocytes with or without HO-1 induction. (A)-Quantitative Real-Time Polymerase Chain Reaction (qRT-PCR) analysis of HO-1 (N=4/group); (B)-Representative image and quantitative analysis of Oil Red O staining (N=4/group). Images were captured using 20X objective lens and the scale bar represents 100 μm (N=8/group); (C)-qRT-PCR analysis of FAS, marker of lipid accumulation (N=4/group); (D)-Triglyceride levels measured by ELISA assay (N=4/group); qRT-PCR analysis of (E)-PGC-1α (mitochondrial biogenesis marker) (N=3–4/group); (F)-IL-6 (inflammatory marker) (N=4/group). For all qRT-PCR *GAPDH* was used as a housekeeping gene. **Note:** (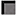)-CTR, (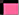)-SnMP, (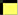)-AngII, (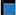)-AngII+CoPP, (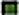)-AngII+CoPP+SnMP; Values represent means ± SEM; (*)-p<0.05 *vs.* CTR; (**)-p<0.01 *vs.* CTR; (##)-p<0.01 *vs.* AngII; (+)-p<0.05 *vs.* AngII+CoPP; (++)-p<0.01 *vs.* AngII+CoPP.

Our results demonstrated a decrease in *HO-1* expression in response to AngII treatment compared to the control group (Figure 1A). The group treated with CoPP showed an increase in *HO-1* expression compared to AngII-treated murine adipocytes. The treatment with SnMP (HO-inhibitor) alone and in combination with CoPP led to an increase in *HO-1* expression. Our data are in agreement with the literature (where it shown that SnMP is a potent inhibitor of HO activity), since a significant increase in *HO-1* expression in SnMP-treated cells has already been described [32]. Analysis of lipid accumulation was evaluated through the relative absorbance of ORO staining in 3T3-L1 adipocytes, demonstrating that AngII treatment induced increased lipogenesis compared to the control group treated with adipogenic media alone and 3T3-L1 adipocytes treated with SnMP alone (Figure 1B). Treatment with only SnMP resulted in significant increase in lipid accumulation compared to the control group. The effects induced by SnMP or AngII treatment alone were significantly attenuated by CoPP-induced antioxidant HO-1 system, an effect reversed by SnMP treatment (Figure 1B). Further, mRNA expression of key protein related to lipid accumulation was evaluated. Our results showed that FAS was significantly upregulated in SnMP-treated cells, and this upregulation was further increased by AngII treatment compared to the control (Figure 1C). CoPP treatment attenuated this increase, which was then reversed by concurrent treatment with SnMP (Figure 1C). In addition, the triglyceride levels were also evaluated, our data demonstrated that the treatment with AngII significantly increased triglyceride levels compared to the controls, and this increase was improved by HO-1 induction (Figure 1D). Simultaneous exposure to SnMP reversed the effect observed with CoPP, leading to an increase in triglyceride levels similar to the increase induced by AngII (Figure 1D). The mRNA expression of the marker of mitochondrial biogenesis, PGC-1α, showed significant downregulation with SnMP treatment alone and AngII treatment compared to the control (Figure 1E). CoPP induction improved this expression, which was then reversed by concurrent treatment with SnMP (Figure 1E). In addition, our data demonstrated a significant increase in the mRNA expression of the inflammatory marker, IL-6, with SnMP treatment alone, and this increase was further exacerbated by AngII treatment compared to the control (Figure 1F). CoPP treatment attenuated this increase, which was then reversed by concurrent treatment with SnMP (Figure 1F).

### Role of AngII on mechanistic interplay between HO-1/SIRT1 axis in 3T3-L1 preadipocytes with or without HO-1 induction

**Figure 2:**
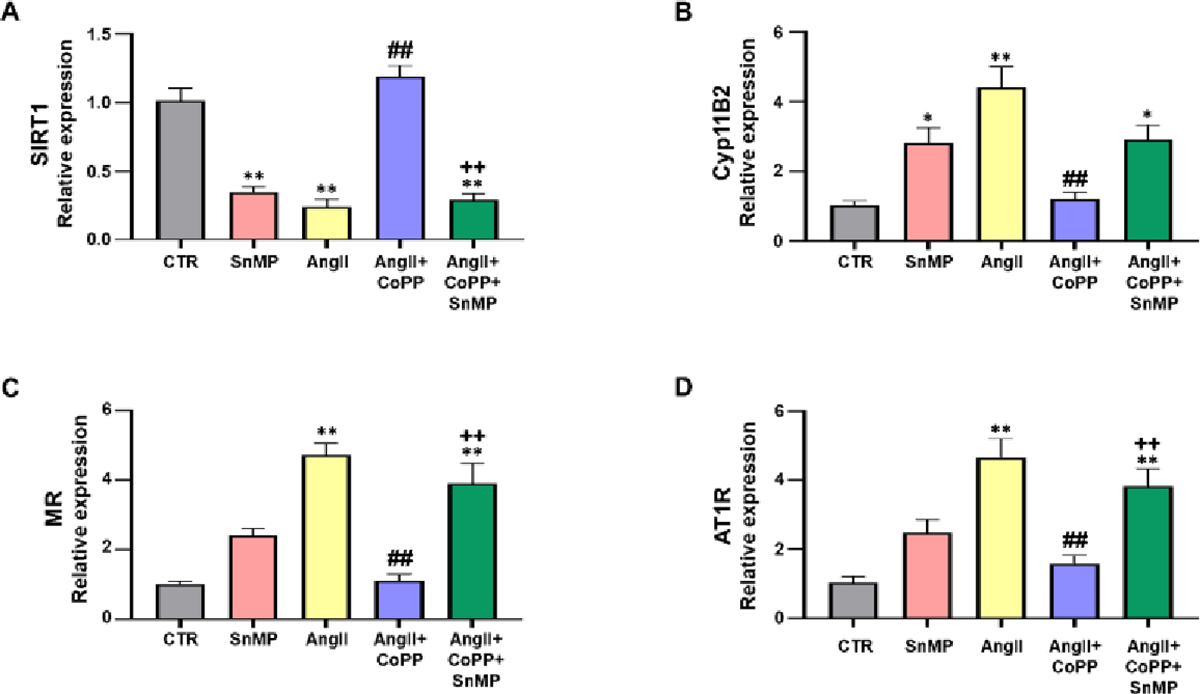
Effect of AngII exposure on 3T3-L1 murine preadipocytes by qRT-PCR for (A)-SIRT1 (N=3–4/group); (B)-*CYP11B2* (N=4/group); (C)-MR (N=4/group); and (D)-AT1R (N=4/group), with *GAPDH* used as a housekeeping gene. **Note:** (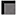)-CTR, (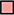)-SnMP, (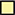)-AngII, (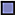)-AngII+CoPP, (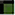)-AngII+CoPP+SnMP; Values represent means ± SEM; (*)-p<0.05 *vs.* CTR; (**)-p<0.01 *vs.* CTR; (##)-p<0.01 *vs.* AngII; (++)-p<0.01 *vs.* AngII+CoPP.

Our subsequent series of experiments aimed to investigate the molecular disruptions induced by AngII that contribute to altered adipocyte phenotype. We performed RT-PCR analyses to evaluate the mRNA expression of *SIRT1*, and our results showed that AngII treatment significantly reduced *SIRT1* expression compared to the control group, this decrease was rescued by HO-1 induction (Figure 2A). However, the improved expression of *SIRT1* was decreased by SnMP treatment. *CYP11B2*, an essential component of the RAAS that contributes to upregulation of MR, also affects adipocyte phenotype. Our results demonstrated that AngII treatment increased the expression of *CYP11B2*, an effect that was reversed by CoPP treatment (Figure 2B). The expression of *CYP11B2* was further increased in the group of murine adipocytes treated with both CoPP and SnMP. Moreover, our results showed an increased expression of *MR* induced by AngII treatment compared to the control group (Figure 2C). This increase in *MR* expression was significantly attenuated by CoPP treatment but reversed by SnMP treatment. Furthermore, we also evaluated the expression of *AT1R*. Our data demonstrated a significant increase in *AT1R* expression with AngII treatment compared to the control group (Figure 2D). CoPP treatment led to a significant decrease in *AT1R* expression, which was subsequently reversed by additional treatment with SnMP.

### Effect of AngII on oxidative stress, inflammation, and adiponectin levels in 3T3-L1 murine preadipocytes with or without HO-1 induction

**Figure 3:**
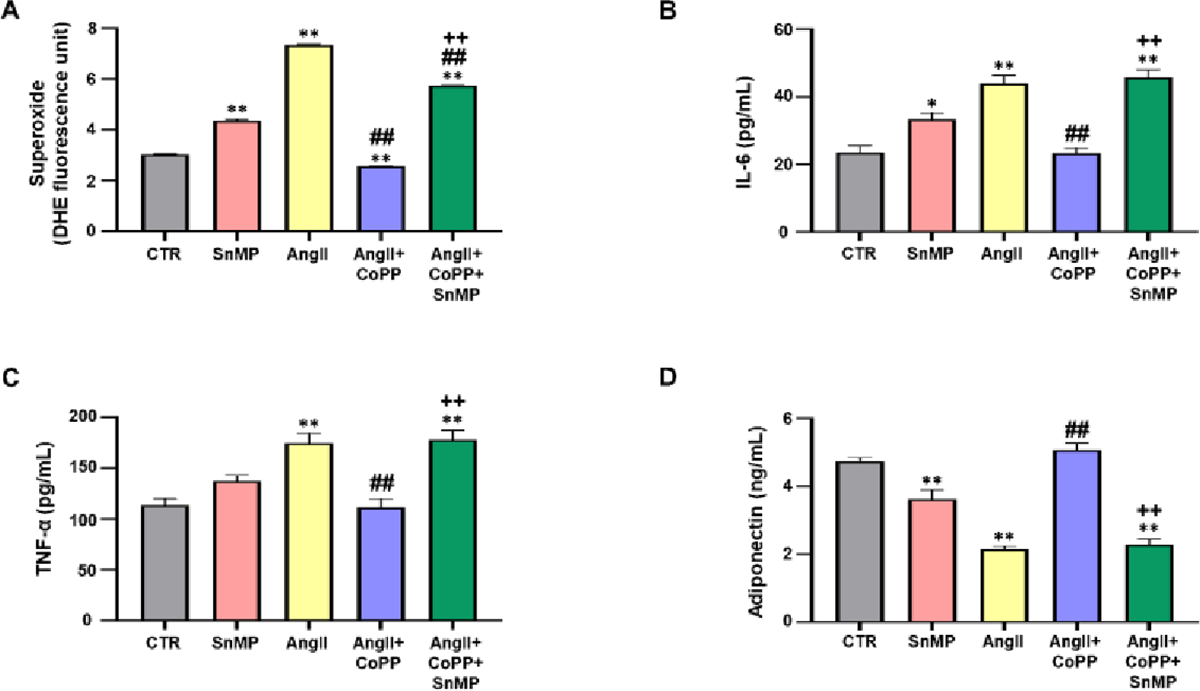
Effect of AngII on oxidative stress, inflammation, and adiponectin levels in 3T3-L1 murine preadipocytes. (A)-Superoxide levels assessed using DHE staining (N=6/group); (B,C)-Levels of inflammatory markers, IL-6 (N=4/group) and TNFα (N=4/group); (D)-Adiponectin levels (N=4/group). **Note:** (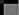)-CTR, (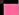)-SnMP, (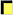)-AngII, (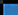)-AngII+CoPP, (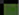)-AngII+CoPP+SnMP; Values represent means ± SEM; (*)-p<0.05 *vs.* CTR; (**)-p<0.01 *vs.* CTR; (##)p<0.01 *vs.* AngII; (++)-p<0.01 *vs.* AngII+CoPP.

The quantification of superoxide levels, an important indicator of Reactive Oxygen Species (ROS) was analyzed by incubating adipocytes with DHE. Our results showed significant upregulation of superoxide in SnMP group and AngII-treated group compared to the control group (Figure 3A). Treatment with CoPP significantly reduced superoxide levels, which were subsequently reversed by SnMP treatment. We also measured the effect of AngII on key markers of the inflammatory process, such as IL-6 and TNF-α. The levels of both inflammatory markers, IL-6 and TNF-α, demonstrated significant increase with AngII treatment compared to the control (Figures 3B and 3C). Furthermore, treatment with CoPP led to a decrease in the levels of inflammatory markers, indicating that this reduction is related to the induction of HO-1. However, the levels of these markers increased in the CM of CoPP-treated cells exposed to SnMP. In addition, the levels of adiponectin were measured in the CM, showing a significant reduction in SnMP and AngII treated cells compared to the control (Figure 3D). However, treatment with CoPP improved adiponectin levels, an effect reversed by the subsequent treatment with SnMP.

### Role of AngII with or without HO-1 induction and SIRT1 knockdown on lipogenesis and FAS levels in 3T3-L1 preadipocytes

**Figure 4:**
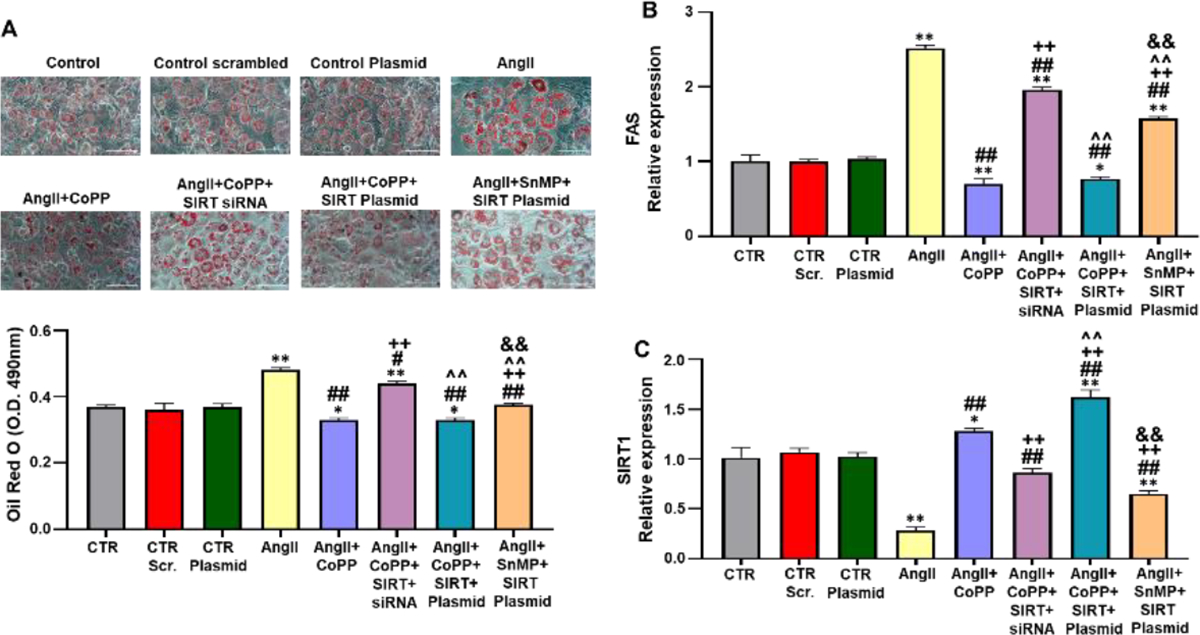
Effect of CoPP with and without SIRT1-siRNA and with and without SIRT1 plasmid on lipid accumulation and FAS expression in 3T3-L1 preadipocytes treated with AngII. (A)-Representative images of adipocytes after Oil Red O (ORO) staining (Upper). Images were captured randomly for qualitative purpose only using 20X objective lens and the scale bar represents 100 μm (N=8/group). The quantitative analysis of ORO staining of independent well (N=4–6/group) (Lower); qRT-PCR analysis of (B)-FAS (N=4–6/group); and (C)-SIRT1 (N=3–4/group), with *GAPDH* used as a housekeeping gene. **Note:** (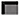)-CTR, (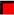)-CTR Scr., (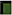)-CTR Plasmid, (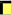)-AngII, (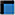)-AngII+CoPP, (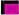)-AngII+CoPP+SIRT+siRNA, (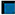)-AngII+CoPP+SIRT+Plasmid, (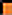)-AngII+SnMP+SIRT+Plasmid; Values represent means ± SEM; (*)-p<0.05 *vs.* CTR; (**)-p<0.01 *vs.* CTR; (#)-p<0.05 *vs.* AngII; (##)-p<0.01 *vs.* AngII; (++)-p<0.01 *vs.* AngII+CoPP; (^^)-p<0.01 *vs.* AngII+CoPP+SIRT+SiRNA; (&&)-p<0.01 *vs.* AngII+CoPP+SIRT+plasmid.

To study the interplay between HO-1 and SIRT1 in mediating their respective actions, we conducted experiments using SIRT1 siRNA and SIRT1 plasmid in 3T3-L1 preadipocytes treated with AngII. Our findings revealed that AngII treatment increased lipid accumulation, as determined by the relative absorbance of ORO staining in 3T3-L1 preadipocytes, and upregulated *FAS* expression (Figures 4A and 4B). However, co-treatment with CoPP significantly attenuated the effects of AngII, resulting in reduced lipid accumulation and *FAS* expression. Simultaneous treatment with CoPP and SIRT1 siRNA led to increased lipid accumulation and *FAS* expression, suggesting that HO-1 acts upstream of SIRT1, and suppression of SIRT1 attenuates the beneficial effects of increased HO-1 levels. Furthermore, we employed *SIRT1* plasmid to investigate whether increased expression of *SIRT1* (in the absence of HO-1 upregulation) could prevent the detrimental effects of AngII on lipid accumulation. Treatment of 3T3-L1 preadipocytes with AngII, SnMP, and SIRT1 plasmid resulted in elevated lipid accumulation and FAS levels compared to cells treated with AngII, CoPP, and SIRT1 plasmid (Figures 4A and 4B). Consistent with our hypothesis, 3T3-L1 preadipocytes treated with AngII, CoPP, and SIRT1 plasmid did not exhibit a significant decrease in lipid accumulation and FAS levels compared to cells treated with AngII and CoPP only, indicating that the activation of *SIRT1* expression is dependent on HO-1. Moreover, our results showed that AngII treatment significantly reduced the mRNA expression of *SIRT1* compared to the control group, whereas CoPP induction increased *SIRT1* expression (Figure 4C). Co-treatment with CoPP and *SIRT1* siRNA led to a decrease in *SIRT1* expression. Notably, the use of SIRT plasmid in the AngII and CoPP-treated group significantly upregulated *SIRT1* expression, an effect that was reversed by subsequent treatment with AngII, SnMP, and SIRT1 plasmid (Figure 4C).

## DISCUSSION

Our research provide evidence that AngII-induced adipocyte dysfunction is accompanied by the suppression of cellular SIRT1, and this effect can be reversed by simultaneous exposure to CoPP, an inducer of the antioxidant HO-1. Experiments using 3T3-L1 preadipocytes revealed that AngII stimulation can modulate different intracellular parameters, leading to an increase of lipogenesis, oxidative stress, release of inflammatory cytokines, and reduced adiponectin levels. Furthermore, our findings demonstrate the upregulation of MR and increased expression of *CYP11B2* in response to AngII treatment, both of which are attenuated by HO-1-SIRT1 axis. In addition, we have shown that the molecular modulation leading an adipocyte phenotype change can be reversed when adipocyte redox balance is restored and *SIRT1* expression is increased through up-regulation of HO-1. Therefore, our results show the beneficial role of the HO-1-SIRT1 axis in adipocytes and provide a basis for considering HO-1 as a potential therapeutic target to improve adipocyte function by attenuating AngII-induced activation in adipocytes.

Oxidative stress plays a vital role in the pathogenesis of metabolic syndrome and associated clinical conditions. Imbalances in redox status and chronic stress contribute to increased adipogenesis and adipocyte dysfunction [23–25,33]. It has been shown that oxidative stress promotes lipid accumulation, modulates key proteins involved in the adipogenesis process, and down-regulates adiponectin levels in adipose tissue [34,35]. In addition, a high oxidative stress is responsible for the suppression of SIRT1, an important cellular survival protein [27,36]. Furthermore, studies have shown that the treatment using antioxidants was found to reduce visceral adiposity, restore metabolic balance, and improve adipocyte function, as evidenced by the increase of adiponectin levels [37,38]. Our findings demonstrate that the up-regulation of the antioxidant system through HO-1 induction attenuates AngII-induced oxidative stress in 3T3-L1 preadipocytes.

This study emphasizes the key role of increased expression of *MR* in the adipocyte dysfunction. MR is a member of the steroid receptor family of ligand activated transcription factors that initiate or suppress the transcription of effector proteins, through several cell signaling pathways [39]. MR has the same binding affinity for aldosterone, cortisol, and corticosterone. Glucocorticoids can also activate MR in most tissues at basal levels and Glucocorticoids Receptor (GR) at stress levels. Inactivation of cortisol and corticosterone by 11β-Hydroxysteroid Dehydrogenase type 2 (11β-HSD2) allows aldosterone to activate MR within aldosterone target cells and limits activation of the GR. Under most conditions, 11β-HSD2 acts as a reductase promoting the inactivation of cortisol to cortisone, which has minimal affinity for MR, protecting the MR from excessive cortisol-induced activation [40]. Previous reports have also highlighted the role of MR in adipose tissue, the excessive activation of this receptor, contributes to several metabolic derangements often observed in obesity, metabolic syndrome and cardiovascular diseases [41–43]. Consistent with these findings, our study demonstrated that treatment of 3T3-L1 preadipocytes with AngII upregulated the expression of *CYP11B2*, which further led to increased *MR* expression. In addition, our data also revealed significant attenuation of these AngII-induced effects through the induction of the HO-1 antioxidant system, which restored *SIRT1* levels, thereby establishing the mechanistic basis of our proposal.

It has already been shown that the induction of HO-1 is able to reduce the production of pro-inflammatory cytokines such as, TNF-α and IL-6 in adipocyte cell culture [44]. Our findings are according to the literature, demonstrating that HO-1 induction leads to increased adiponectin levels in 3T3-L1 preadipocytes. Adiponectin is an adipocyte-derived hormone that has been shown to present a positive impact on triglyceride levels. In addition, decreased levels of adiponectin are closely linked to increased oxidative stress [45,46]. The data obtained in this study, suggest that AngII-induced downregulation of adiponectin contributes to altered adipocyte phenotype, while the protective effect of HO-1 increases adiponectin levels, promoting healthier adipocytes. Furthermore, the upregulated *HO-1* expression induced by CoPP has the ability to reprogram the altered adipocyte phenotype into a healthy state by improving oxidative stress, reducing the release of inflammatory cytokines, decreasing lipid accumulation, and increasing adiponectin levels.

**Figure 5:**
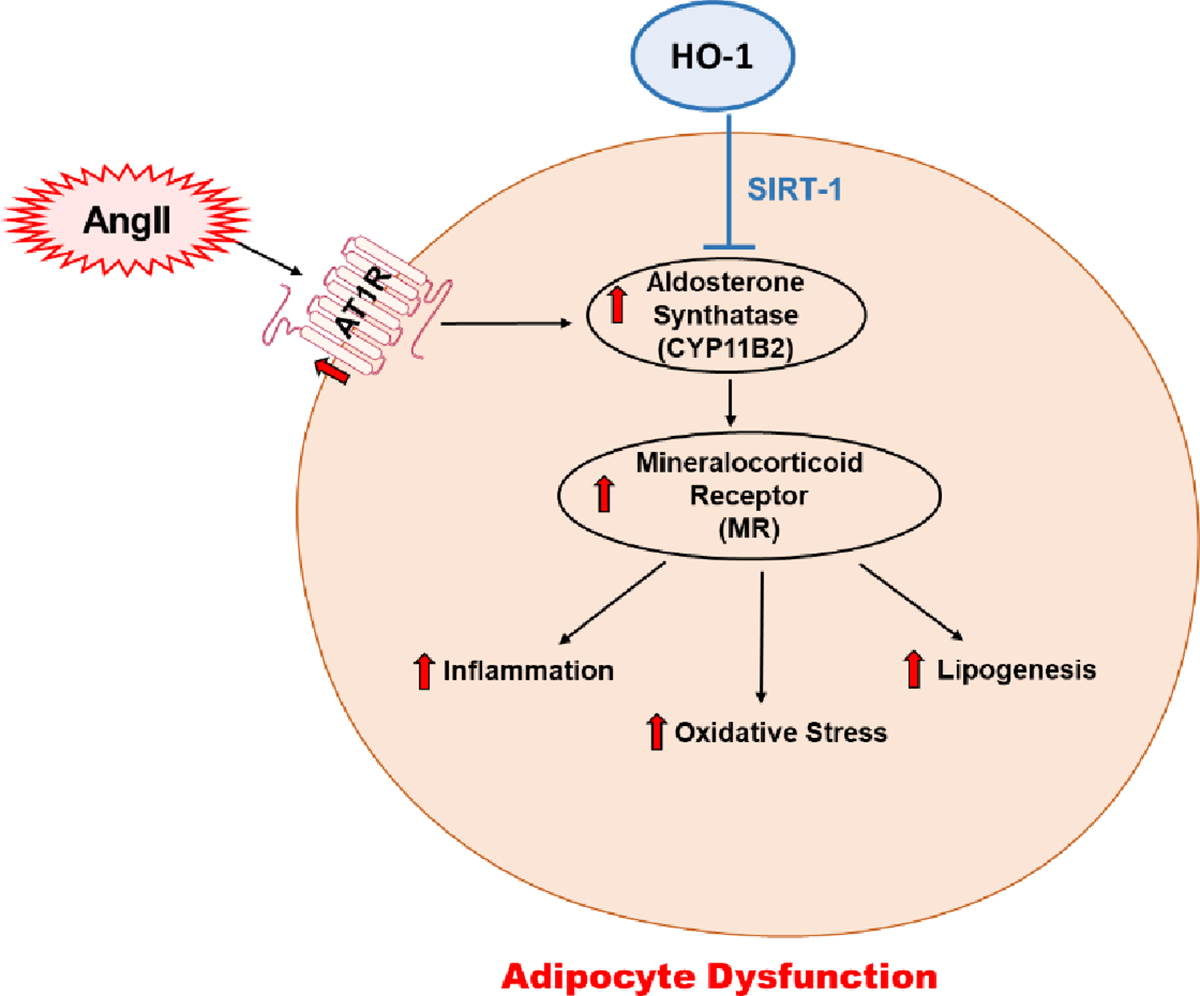
Schematic diagram depicting AngII-mediated phenotypic modifications in adipocytes reversed through HO-1-dependent SIRT1 rescue in 3T3-L1 murine adipocytes.

Our data reveals a mechanistic association between exposure to AngII and SIRT1, a member of NAD-dependent deacetylase family that is a significant regulator of energy metabolism as well as of many survival functions [27,47]. In addition, it has been shown that oxidative stress can inhibit SIRT1 levels [48]. In our study we showed that the oxidative stress induced by AngII leads to the downregulation of SIRT1 in adipocytes, and interestingly, the 3T3-L1 preadipocytes treated with CoPP was able to rescue *SIRT1* expression and suppression of *MR*, suggesting the protective effect of HO-1 on cellular SIRT1 (Figure 5).

## CONCLUSION

In conclusion, the upregulation of AngII modulates oxidative and inflammatory pathways in adipocytes, promoting phenotypic and molecular changes through inhibition of the HO-1-SIRT1 axis. The data obtained in this study show that the rescue of SIRT1 is dependent on HO-1, which effectively reverses the molecular and pathological effects of the AngII cascade in 3T3-L1 preadipocytes. These findings are summarized in a scheme, showing the AngII-mediated increase in *CYP11B2*, which subsequently triggers inflammation, oxidative stress, and lipogenesis. However, HO-1-mediated rescue of SIRT1 leads to attenuation of the effects listed above, promoting an improvement in adipocyte phenotype in addition to molecular changes. Our study has a novel clinical implication for patients with metabolic disorders dependent on the RAAS, as well as those with secondary RAAS activation observed in conditions such as obesity. The findings highlight the therapeutic potential of HO-1 induction as a complementary therapy to alleviate adipose tissue dysfunction, reduce systemic inflammation, enhance adiponectin levels, and restore metabolic balance in these patient populations.

## Abbreviations:

RAAS: Renin-Angiotensin-Aldosterone System
AngII: Angiotensin II
VAT: Visceral Adipose Tissue
AGT: Angiotensinogen
MR: Mineralocorticoid Receptor
SIRT1: Sirtuin 1
HO: Heme Oxygenase
CoPP: Cobalt Protoporphyrin
SnMP: Tin Mesoporphyrin
ROS: Reactive Oxygen Species
DHE: Dihydroethidium
TNF-α: Tumor Necrosis Factor α
IL-6: Interleukin 6
FAS: Fatty Acid Synthase
PPARγ: Peroxisome Proliferator-Activated Receptor γ
HSD2: 11β-Hydroxysteroid Dehydrogenase type 2
